# Correction: Resistance to DDT and Pyrethroids and Increased kdr Mutation Frequency in *An. gambiae* after the Implementation of Permethrin-Treated Nets in Senegal

**DOI:** 10.1371/annotation/855f784c-b8db-449c-9de8-83b6404a049d

**Published:** 2012-04-26

**Authors:** Mamadou O. Ndiath, Seynabou Sougoufara, Abdoulaye Gaye, Catherine Mazenot, Lassana Konate, Ousmane Faye, Cheikh Sokhna, Jean-Francois Trape

There was a spelling error in the sixth author's name. The correct spelling is Ousmane Faye. 

